# Osteosarcoma: Cells-of-Origin, Cancer Stem Cells, and Targeted Therapies

**DOI:** 10.1155/2016/3631764

**Published:** 2016-06-05

**Authors:** Ander Abarrategi, Juan Tornin, Lucia Martinez-Cruzado, Ashley Hamilton, Enrique Martinez-Campos, Juan P. Rodrigo, M. Victoria González, Nicola Baldini, Javier Garcia-Castro, Rene Rodriguez

**Affiliations:** ^1^Haematopoietic Stem Cell Laboratory, The Francis Crick Institute, London WC2A 3LY, UK; ^2^Central University Hospital of Asturias (HUCA) and Institute of Oncology of Asturias (IUOPA), 33011 Oviedo, Spain; ^3^Complutense University of Madrid, 28040 Madrid, Spain; ^4^Orthopaedic Pathophysiology and Regenerative Medicine Unit, Rizzoli Orthopaedic Institute, 40136 Bologna, Italy; ^5^Department of Biomedical and Neuromotor Sciences, University of Bologna, 40123 Bologna, Italy; ^6^Cellular Biotechnology Laboratory, Institute of Health Carlos III (ISCIII), Majadahonda, 28220 Madrid, Spain

## Abstract

Osteosarcoma (OS) is the most common type of primary solid tumor that develops in bone. Although standard chemotherapy has significantly improved long-term survival over the past few decades, the outcome for those patients with metastatic or recurrent OS remains dismally poor and, therefore, novel agents and treatment regimens are urgently required. A hypothesis to explain the resistance of OS to chemotherapy is the existence of drug resistant CSCs with progenitor properties that are responsible of tumor relapses and metastasis. These subpopulations of CSCs commonly emerge during tumor evolution from the cell-of-origin, which are the normal cells that acquire the first cancer-promoting mutations to initiate tumor formation. In OS, several cell types along the osteogenic lineage have been proposed as cell-of-origin. Both the cell-of-origin and their derived CSC subpopulations are highly influenced by environmental and epigenetic factors and, therefore, targeting the OS-CSC environment and niche is the rationale for many recently postulated therapies. Likewise, some strategies for targeting CSC-associated signaling pathways have already been tested in both preclinical and clinical settings. This review recapitulates current OS cell-of-origin models, the properties of the OS-CSC and its niche, and potential new therapies able to target OS-CSCs.

## 1. Introduction

OS is a malignant neoplasm in which the neoplastic cells produce bone and is the most frequent primary sarcoma of the skeleton. The tumor is primary when the underlying bone is normal and secondary when the bone is altered by conditions, such as prior irradiation, coexisting Paget disease, infarction, or other disorders. It has a bimodal age distribution with most cases developing between the ages of 10–16 years and a second smaller peak in older adults (30% of cases in patients over 40 years) [1]. In addition, OS is the most common radiation-induced sarcoma. It has an unknown etiology, although there is an increased incidence of primary OS associated with several genetic syndromes such as Li-Fraumeni, hereditary retinoblastoma, and Rothmund Thomson (see below).

Primary OS may arise in any bone, although the vast majority originate in the long bones of the extremities, especially the distal femur (30%), followed by the proximal tibia (15%), and proximal humerus (15%), which represent sites containing the most proliferative growth plates. Within long bones, the tumor is usually (90%) located in the metaphysis and arises as an enlarging and palpable mass, with progressive pain [2].

The hallmark diagnostic feature of OS is the detection of osteoid matrix produced by the neoplastic cells. However, the most common type of OS, conventional OS, has a very broad spectrum of histological appearances and is subclassified according to the predominant type of stroma (osteoblastic, chondroblastic, fibroblastic, giant cell rich, etc.), although this subclassification has no prognostic relevance [1].

At present, surgery with chemotherapy is the first-line treatment for most OS [3]. Almost all patients receive neoadjuvant intravenous combinational chemotherapy (doxorubicin and cisplatin with or without methotrexate) as initial treatment. Surgical resection of the primary tumor with adequate margins is an essential component of the curative strategy for patients with localized OS. If complete surgical resection is not feasible or if surgical margins are inadequate, radiation therapy may improve the local control rate. The postoperative chemotherapy regimen usually depends on the extent of tumor necrosis observed [1, 3].

Advances in the clinical management of OS have led to a significant increase in 5-year survival rates, which in most centers now largely exceed 50%. However, survival rates for patients presenting with metastatic and recurrent disease have historically remained essentially unchanged with a survival rate below 20%, highlighting the need for a better understanding of the disease leading to the development of novel therapies [4].

## 2. Genomics of OS

OS is characterized by the presence of complex karyotypes indicative of severe chromosomal instability. This accumulation of barely recurrent genetic alterations hinders the identification of OS-driver genes. A powerful causal-effect relation between specific gene alterations and OS initiation came from studies of human hereditary disorders characterized by a predisposition to the development of OS [5, 6]. The functional validation of these genomic alterations as driver events was confirmed in mouse models [5, 7]. The strongest genetic association for sporadic and hereditary OS is with the retinoblastoma (*RB*) and the* P53* tumor suppressor genes; meanwhile other relevant alterations include mutations in other cell cycle regulators, oncogenes, and DNA helicases [5, 6].

Li-Fraumeni and hereditary retinoblastoma syndromes are caused by heterozygous germ-line mutations of* P53* and* RB*, respectively, and patients presenting with these disorders have a higher predisposition to a range of cancers, including OS [8, 9]. Importantly, mutations in* P53* and/or* RB* genes and other components of their pathways are also common in sporadic OS, suggesting a relevant role for alterations in these tumor suppression genes or their related signaling pathways in OS development [5, 6, 10, 11]. On this basis, several* P53* and/or* RB*-deficient mouse models have been developed to model sarcomagenesis [5, 12]. The most productive OS models have been developed using conditional mesenchymal/osteogenic lineage-restricted mutation of* P53* and* RB* (see below). These models indicate that* P53* inactivation is an initiating event in OS [13–15]. On the other hand, the depletion of* RB* alone was not sufficient to induce sarcoma formation in mice. Notably,* RB* mutation strongly reduced the latency required for sarcoma formation in* P53*-deficient mice, although it decreased the proportion of OS formed [13, 15]. It was reported that RB is needed to promote the osteogenic differentiation program of mesenchymal stem/stromal cells (MSCs) [16] and, therefore, it could be speculated that* RB* mutations synergize with* P53* inactivation in OS formation only when mutations occur in osteogenic-committed cell types; meanwhile it could favor other sarcoma phenotypes when mutated in more immature cell types (see below).

Other genes involved in P53 or RB signaling have also been found to be mutated in sporadic OS [6, 17]. For example, the* INK4A/ARF* locus, which encodes for* P16INK4A* and* P19ARF* genes, is deleted in approximately 10% of OS [18, 19]. P16INK4A and P19ARF proteins contribute to the stabilization of RB and P53 proteins through the inhibition of cyclin-dependent kinase 4 (CDK4) and mouse double minute 2 homolog (MDM2) repressors, respectively [20]. Interestingly, the region 12q13, containing* CDK4* and* MDM2*, is amplified in up to 10% of OS [6, 21]. In addition, the absence of expression of* P16INK4A* correlated with a decreased survival in pediatric OS, while the amplification of* MDM2* has been associated with the development of metastases in OS [6, 22]. The amplification and/or increased expression of other cell cycle genes, such as Cyclins* D1* and* E*, have also been reported in OS, further highlighting an important role of defective cell cycle regulation in OS development [17, 23].

Several oncogenes like* C-FOS*,* C-JUN,* and* C-MYC* also play a role in OS development. C-FOS, C-JUN N-terminal kinase, and C-JUN were found elevated in OS and its expression and activation were associated with the progression of human OS [24–26]. Transgenic mice overexpressing* C-FOS* developed OS, further suggesting a role in OS pathogenesis [27].* C-MYC* amplification was found in sporadic OS and OS associated with Paget's disease [28, 29] and, clinically, high C-MYC expression correlates with worse outcome in OS patients [30].

A recent study using a* Sleeping Beauty* transposon-based forward genetic screen in mice, with or without somatic loss of* P53* restricted to committed osteoblast progenitors, identified 36 putative protooncogenes and 196 potential tumor suppressor genes. Among these OS-driver candidates the protumorigenic role of* PTEN* and the axon guidance genes* SEMA4D* and* SEMA6D* were functionally validated. Moreover, this study highlighted an enrichment of genes involved in PI3K-AKT-mTOR, MAPK, and ERBB signaling cascades [51]. Confirming the heterogeneity of OS, an exome sequencing-based study showed that identified candidate OS-driver genes were associated with the development of a small set of tumors, suggesting that multiple oncogenic pathways drive the characteristic chromosomal instability during OS development. However, the overall mutation signatures in these tumors were reminiscent of BRCA1/2 deficient tumors, a finding with possible therapeutic implications [52]. Comparative genomic hybridization analysis combined with gene expression data also resulted in the identification of genomic alterations associated with a small proportion of OS, which may play a role in the OS pathogenesis. For instance, the cell division-related genes* MCM4* and* LATS2*, the antiapoptotic genes* BIRC2* and* BIRC3,* and other genes including* CCT3*,* COPS3*, and* WWP1* were reported to be found as potential OS drivers [53–55]. Likewise, genomic analysis indicated that ossification factor genes such as* MET*,* TWIST,* and* APC* are frequently mutated in pediatric high-grade OS and these alterations correlated to a worse outcome, thus suggesting a role in OS development [56].

Other genetic and epigenetic alterations likely involved in OS pathogenesis include mutations in* RECQL4* DNA helicase associated with the OS-predisposing Rothmund Thomson syndrome, amplification/mutation in the osteogenic factor RUNX2, loss of heterozygosity of FGFR2 and BUB3, enhanced telomerase activity, deletion of* PRKAR1A*, and reduced expression of* WWOX* or hypermethylation of* HIC1* in P53 mutated tumors among others [5, 7, 57–61].

## 3. Cell-of-Origin for OS

**Figure 1:**
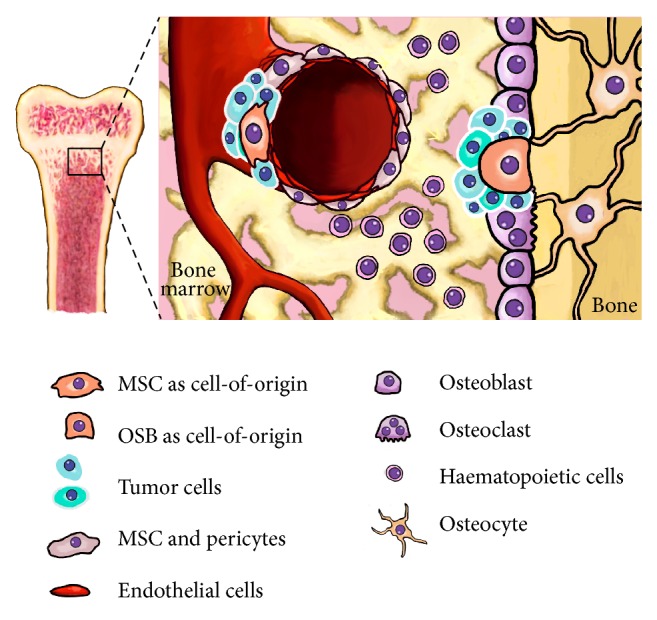
Cell-of-origin for OS. The figure shows the most relevant cell types present in the bone microenvironment. MSCs, represented in a perivascular niche, and their derived cell types along the osteogenic lineage, such as the osteoblasts (OSB), are strong candidates to acquire the first cancer-promoting mutations and initiate OS formation.

The cell-of-origin concept refers to the normal cell type that acquires the first cancer-promoting mutations and initiates tumor formation [62]. During the last 10 years mounting evidence has placed MSCs and/or their immediate lineage progenitors as the most likely cell-of-origin for many types of sarcomas including OS [12, 63, 64] (Figure 1). Both translocation- and non-translocation-associated sarcomas have been modeled by introducing relevant mutations into MSC [12, 64–66]. In the case of OS, most of the cell-of-origin models are based on mutated* P53*, alone or in combination with* RB* inactivation, in the mesenchymal/osteogenic lineage of mouse models or in murine MSC [63]. By crossing mice with conditional (floxed) alleles of* P53* and/or* RB* with mice that have the* CRE* recombinase gene under the control of different tissue-restricted promoters, several groups were able to generate OS development* in vivo*. Thus, the inactivation of* P53* and/or* RB* in early mesenchymal progenitors of embryonic limb buds through* PRX1*-driven* CRE* expression (*PRX1-CRE*) resulted in sarcoma development, presenting an OS incidence of 60% in* P53* ^−/−^ mice and 20–30% in* P53* ^−/−^ *RB* ^−/−^ mice, where most of the alternate tumors formed poorly differentiated soft tissue sarcomas [14, 16]. Meanwhile, the inactivation of these cell cycle regulators in osteoblast precursors [*OSX1* (Osterix)-*CRE*] resulted in a higher incidence of OS formation in both* P53* ^−/−^ (100%) and* P53* ^−/−^ *RB* ^−/−^ mice (between 53 and 100%) [13, 15, 16]. Similarly, ShRNA-mediated depletion of* P53*, together with CRE-mediated inactivation of* RB* in osteoblast precursors (*OSX1* restricted), resulted in a delayed and consistent formation of OS, presenting a higher degree of osteoblastic differentiation than other CRE-based models [67]. Within the osteogenic differentiation lineage, targeting of* P53* in mature osteoblasts, using* COL1A1-* (collagen-1-alpha-1-) driven* CRE* expression to target* P53* or* OCN-* (Osteocalcin-) driven expression of SV40-T antigen to inactivate* P53* and* RB*, also resulted in high OS formation incidence (85–100%) [14, 68]. Moreover, another study using a SV40 immortalized murine osteocyte cell line suggests that fully differentiated osteocytes may also serve as an OS-initiating cell [69]. Besides P53 deficiency-based OS models, it has been proven that the expression of the intracellular domain of NOTCH1 (*NICD*), leading to constitutive NOTCH activation, in osteoblasts (*COL1A1*-driven expression) was sufficient to induce the formation of bone tumors, including OS [70]. Moreover, NOTCH activation combined with loss of P53 synergistically accelerates OS formation. Notably, the activation of NOTCH in mesenchymal progenitors or in osteoblast precursors produces embryonic lethality [70]. Similarly, mice with upregulated Hedgehog (HH) signaling in mature osteoblasts with a* P53* heterozygous background developed OS with high penetrance [71].

These results support the concept that OS originates in the population of cells that undergoes osteoblast commitment rather than in immature MSC. Nevertheless, these experiments also show that, although presenting at a lower incidence, early mesenchymal progenitors targeted with relevant mutations could also initiate OS formation, most likely influenced by certain microenvironment signals. In this regard, the comparison in a single study of the OS formation ability of* P53/RB*-disrupted immature MSC (*PRX1-CRE*) and osteoblast committed cells (*COL1A1-CRE* and* OCN-CRE*) confirmed that all types of cells were able to initiate OS formation and showed that the level of osteoblastic differentiation of tumors did not correlate with the degree of differentiation of the cell-of-origin, suggesting that epigenetic dedifferentiation mechanisms could be active in mature osteoblasts during osteosarcomagenesis [72]. The fact that early progenitors might represent a cell-of-origin for OS is also strengthened by the observation of different histological subtypes, which may reflect the ability of these progenitors to undergo other differentiation pathways besides osteogenesis.

Studies using murine MSC containing CRE-inactivated* P53* and/or* RB* alleles also reveal relevant clues about the nature of the OS-initiating cell and the factors conditioning their sarcomagenic potential.* P53* ^−/−^ and* P53* ^−/−^ *RB* ^−/−^ adipose-derived-MSC (ASC) or BM-derived-MSC (BM-MSC) give rise to leiomyosarcoma-like tumors when injected subcutaneously into immunodeficient mice [72–74]. Otherwise, when BM-MSCs were differentiated along the osteoblastic lineage before CRE-mediated deletion of* P53* and* RB*, they generated OS-like tumors upon subcutaneous injection into immunodeficient mice, whereas* P53* ^−/−^ *RB* ^−/−^ ASC-derived osteogenic progenitors did not display tumorigenic potential [74]. These data highlight the differences in the sarcomagenic potential of MSC from different tissues and indicate that a certain level of osteogenic differentiation of BM-MSC is needed for the development of the OS phenotype. Nevertheless, orthotopic (intrabone or periosteal) inoculation of* P53* ^−/−^ and* P53* ^−/−^ *RB* ^−/−^ BM-MSC and ASC undifferentiated MSC consistently generated osteoblastic OS displaying human OS radiographic and histological features alongside metastatic potential. Importantly, all the histological and radiological OS-related features were less evident or completely lost in the areas of the tumor distant from the recipient bone, thus demonstrating that bone microenvironmental signals play a role in osteogenic differentiation and sarcomagenesis by defining the sarcoma phenotype [75]. In addition, an ectopic model to assay osteosarcomagenesis that relies on the use of* P53* ^−/−^ *RB* ^−/−^ MSC embedded in hydroxyapatite/tricalcium phosphate ceramics also demonstrates a relevant contribution of bone microenvironmental factors, like bone morphogenic protein 2 (BMP2) and calcified substrates, in the acquisition of the OS phenotype [75].

Furthermore, evidence of undifferentiated MSC as cell-of-origin for OS comes from the introduction of other oncogenic events into undifferentiated BM-MSC, like the expression of* C-MYC* in a* P16INK4A* ^−/−^ *P19ARF* ^−/−^ genetic background or the aneuploidization accompanied by the loss of the INKAA locus, resulting in OS development [76, 77]. Additionally, similar gene expression signatures were found between human OS samples and undifferentiated MSC or osteogenic-committed MSC [78], suggesting that OS may develop from both osteogenic progenitors and undifferentiated MSC.

Finally, extraskeletal OS is a very rare type of soft tissue mesenchymal neoplasm that produces osteoid. It could be speculated that, rather than BM-MSC, extraskeletal OS could be initiated by MSCs from soft tissues (muscle-derived MSC, ASC, etc.) presenting mutations that favor osteogenic differentiation and/or influenced by pathologically osteogenic signals from the microenvironment [79]. In this regard this type of tumors could be related to fibrodysplasia ossificans progressiva, a rare genetic disorder of connective tissue characterized by the presence of activating mutations in the ACVR1 gene, which encode a BMP type I receptor [80].

Overall, the most likely situation is that either MSC-derived osteogenic progenitors or undifferentiated MSC may represent the cell-of-origin for OS under the influence of proper microenvironmental or epigenetic signals.

## 4. Osteosarcoma Cancer Stem Cell

Experimental evidence supports the notion that sarcomas are hierarchically organized and sustained by a subpopulation of self-renewing cells that can generate the full repertoire of tumor cells and display tumor reinitiating properties [12, 81–83]. In the most likely scenario, these CSC subpopulations emerge after the accumulation of further epigenetic and/or genetic alterations in a cell within the aberrant population, initially generated by the cell-of-origin [62], that is, MSC-derived cell types.

Several methods have been developed to isolate and/or enrich subpopulations with stem cell properties within the tumors [82–85]. The isolation of OS-CSC was first achieved on the basis of their ability to form spherical and clonal expanding colonies (sarcospheres) in anchorage-independent and serum-starved conditions [86–88]. This sarcosphere formation may be further improved by reproducing the hypoxic conditions of tumor microenvironment [89]. In addition, OS-CSCs are commonly isolated by sorting cells according to the expression of specific surface markers associated with stemness and/or tumorigenesis. For example, CD133^+^ [90–92], STRO1^+^ CD117^+^ [93], and CD271^+^ populations [94] sorted from OS cell lines demonstrated CSC-like features. Other methods used to isolate OS-CSCs include the identification of a “side population” of cells able to exclude fluorescent dyes, alone or in combination with surface markers like CD248 [95, 96]; the sorting of cells with aldehyde dehydrogenase 1 (ALDH1) activity [97, 98]; the tracking of subpopulations that express pluripotency-associated genes, such as* OCT-4* [99]; the enrichment of subpopulations with high telomerase activity [100, 101]; or the long-term treatment with chemotherapy [102, 103]. Reinforcing their expected mesenchymal progenitor origin, many of these sarcoma-initiating cells express MSC markers [86, 93, 99, 104] and retain* in vitro* differentiation properties, giving rise to adipogenic, chondrogenic, and osteogenic lineages [86, 104]. These CSC commonly show increased expression of the pluripotent stem cell markers OCT3/4, NANOG, and SOX2 [87, 89, 105, 106]. Remarkably, SOX2 has been reported to identify a population of CSC in OS required for self-renewal and tumorigenesis [107]. Importantly, CSCs isolated from OS are able to self-renew and sustain tumor generation in serial transplantation experiments and are associated with metastasis and drug resistance [89, 93, 96, 98, 105, 107]. This increased chemoresistance of CSC subpopulations has been associated with an increase in the DNA repair ability, with an inhibition of the apoptotic signaling, with increased levels of lysosomal activity due to the overexpression of vacuolar ATPse [108], and, specially, with a gain in the drug efflux capacity due to the overexpression of the ABC transporters [96, 102, 105, 109–111]. In this line, the inhibition of the ABC transporters is able to sensitize OS-derived sarcosphere to doxorubicin [112]. Therefore, it is clear that OS-CSCs possess specific properties, which make them more resistant to therapies.

**Figure 2:**
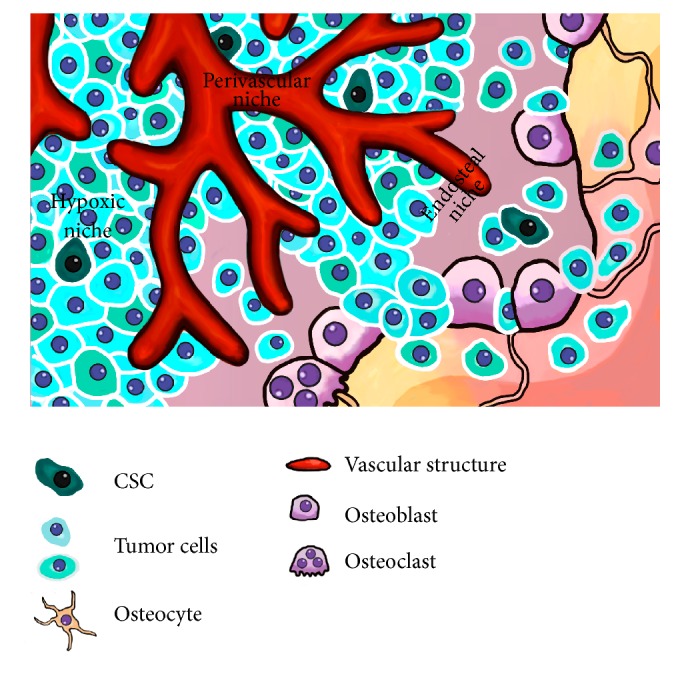
OS-CSC niches. The figure represents possible niches for CSCs in OS. Suggested locations for CSCs are the perivascular niche, the endosteal niche, and areas of poor vascularization (hypoxic niche).

Similar to normal stem cells, microenvironmental niches may play a role in OS-CSC regulation [113]. In this regard, many bone microenvironment signals, including those mediated by fibroblastic growth factor (FGF), transforming growth factor *β* (TGF-*β*), insulin-like growth factor 1 (IGF1), BMP, vascular endothelial growth factors (VEGF), hypoxia inducible factors (HIF1), wingless-type MMTV integration site family (WNT), HH, or NOTCH among others, are deregulated in OS and seem to be involved in the regulation of their self-renewal, differentiation, growth, drug resistance, and/or metastatic potential of OS-CSC subpopulations (reviewed in [2]). Three types of niches within the bone microenvironment where these signaling pathways are particularly active could be inferred to be the location OS-CSCs: the perivascular niche, the hypoxic niche, and the endosteal niche (Figure 2). OS is a highly vascularized tumor and as OS-CSCs seem to arise from MSC it is likely that OS-CSCs may be located in the same perivascular niche already well-described for normal MSCs. Besides providing stemness signals, this perivascular location would favor migration and metastasis of CSC [84, 114]. On the other hand, the bone is a hypoxic environment and hypoxia-induced signaling is a key environmental factor involved in stemness maintenance, OS progression, and drug resistance and therefore could constitute a suitable niche for OS-CSCs [2, 115]. Finally, the endosteal niche is a rich environment where tumor cells interfere with the bone remodeling process, establishing a “vicious cycle” that favors osteoclast-mediated osteolysis and the subsequent release of calcium and growth factors (FGF, TGF-*β*, IGF1, BMP, etc.), which support stem and tumorigenic properties [2, 116, 117].

## 5. Cancer Stem Cell Targeted Therapies in OS

Studies concerning the molecular biology of cancer are now promoting the identification of new potential therapeutic targets with molecular rationale able to target OS. As a result, therapies targeting altered signaling are being thoroughly tested in several clinical trials [58, 118–120] (Table 1). These strategies included the targeting of the signaling mediated by receptor tyrosine kinases (EGFR, VEGFR, IGF1R, HER2, or PDGFR), mTOR, or WNT/*β*-catenin. In addition, since OS-CSCs reside within the bone microenvironment and this a key factor in the regulation of tumor homeostasis, therapies directed against microenvironmental niche factors could contribute to the improvement of clinical response [2]. Therefore, several therapeutic strategies have been developed to target the role of the tumor-promoting osteoclast activity [31–33, 121], to reduce the vascularization of tumors [34, 122] and to enhance the immune response against tumors [123–126] (Table 1).

As seen before, OS-CSCs are resistant to most conventional treatments like radiation and chemotherapy and are, therefore, responsible for tumor relapses and metastasis. Hence, in addition to the proposed therapies directed against specific signaling and/or tumor niches, there is a need for developing and testing therapies able to target CSC subpopulations in OS. Below, we reviewed current work reporting specific antitumoral activity on OS-CSC subpopulations or CSC-related features (Table 2).

Broad genomic, metabolomic, and proteomic analyses have been useful to better characterize OS-CSC and therefore define potential OS-CSC-specific therapeutic targets with molecular rationale [58, 127, 128]. Among the reported altered signaling pathways with therapeutic implications, nuclear factor *κ*B (NF-*κ*B) is activated in radioresistant subpopulations of OS cell lines, and these subpopulations could be sensitized to radiation by parthenolide, an inactivator of NF-*κ*B [129]. Another NF-*κ*B inhibitor, BRM270, specifically targeted a multidrug resistant OS stem-like cell population by increasing their apoptosis rate and thereby reducing tumorigenic potential [130]. Phosphatidylinositol-3-kinase (PI3K) is also an interesting therapeutic target due to its high mutation frequency and its role in regulation of proliferation, cell cycling, survival, and apoptosis. BYL719, a specific PI3K*α* inhibitor, induces cell cycle arrest and inhibition of cell migration in OS cells, and, therefore, has been postulated to be useful for multidrug therapeutic approaches [35]. Moreover, another PI3K inhibitor, LY294002, induces cell cycle arrest and apoptosis in OS-CSC [131].

Developmental signaling pathways like WNT and NOTCH, which are highly involved in the regulation of stemness and differentiation, have also been reported to play a role in OS development [2, 37, 70]. In OS cell lines, the inactivation of NOTCH and WNT pathways resulted in sensitization to chemotherapeutic drugs [36]. In addition, aberrant active WNT/*β*-catenin signaling has been described in the OS-CSC population and has been associated with SOX2 overexpression and tumorigenicity [38, 132]. On the other hand, the WNT-antagonist Dickkopf proteins 1 (DKK1) has been proposed to enhance protumorigenic properties in OS, in part, through the upregulation of the stress response enzyme and CSC marker ALDH1A1 [133]. In this case, the inhibition of the canonical WNT pathway by DKK1 leads to the activation of noncanonical JUN-mediated WNT pathways, which mediate the induced tumorigenic effects. Likewise, NOTCH signaling has been associated with ALDH activity and increased metastatic potential in OS cells [39]. Therefore, a number of different therapies have been assayed to target WNT or NOTCH pathways via downregulation, inactivation, or silencing techniques [2, 37, 40] (Table 1). Interestingly, curcumin, a natural product that shows high antitumoral activity against OS cells, is a WNT/*β*-catenin antagonist whose antitumor activity seems to be mediated through the inactivation of NOTCH1 signaling, thereby linking both signaling pathways [41]. In addition, TGF-*β* is also a well-known regulator of bone biology that plays a relevant role in OS development [42]. The blocking of TGF-*β* signaling using the natural TGF-*β*/SMAD signaling inhibitor SMAD7, the inhibitor of TGF-*β* receptor complexes SD-208, or the natural alkaloid halofuginone hindered OS progression. These treatments reduced* in vivo* tumorigenic potential of OS cell lines, repressed tumor-associated bone remodeling, and inhibited the development of metastasis [43, 44]. Moreover, TGF-*β* signaling activation has been involved in the induction of stemness, tumorigenicity, metastatic potential, and chemoresistance in nonstem OS cells, and, conversely, the blocking of this signaling resulted in the inhibition of this dedifferentiation process of nonstem cell populations, thereby highlighting TGF-*β* as a potential therapeutic target [45].

Not surprisingly, microRNAs (miRNAs) are extensively related to OS development [46]. In a CSC context, a list of 189 miRNAs that are differentially expressed in OS-CSC has been reported [127]. Some of these miRNAs, such as miR-382 and miR-29b-1, were significantly decreased in human OS and their overexpression resulted in a decrease in CSC properties, metastatic potential, or chemoresistance, thus suggesting that these miRNAs could constitute novel therapeutic strategies to target OS-CSC [47, 48]. On the other hand, high levels of miR-133a and CD133 correlated with poor prognosis in OS and the inhibition of miR-133a associated with chemotherapy suppressed lung metastasis and prolonged survival in preclinical models of OS [49]. Moreover, other miRNAs like miR-215 and miR-140 have also been related to OS chemoresistance [50, 134]. In addition, a recent report shows that the overexpression of the novel long noncoding RNA HIF2PUT, involved in the regulation of HIF-2*α* expression, markedly inhibited proliferation, migration, and stem cell features in OS cells, thus providing a proof of principle for testing HIF2PUT in future therapeutic strategies [135].

Several natural compounds with reported antitumoral activity in OS have recently been shown to demonstrate specific inhibitory effects in OS-CSC (Table 2). Thus, besides inhibiting proliferation, invasion, and metastatic potential in OS cells, the hypoglycemic agent metformin also induces a marked reduction of the self-renewal and differentiation potential of CSC subpopulations and sensitizes OS cell to cisplatin [136, 137]. In a similar way, bufalin inhibits the differentiation and proliferation of OS-CSC through a mechanism regulated by miR-148a [138, 139]. Also, the polyether ionophore antibiotic salinomycin has demonstrated specific antitumoral activity against OS-CSC [140]. Moreover, salinomycin-loaded nanoparticles conjugated with CD133 aptamers highly increase the therapeutic effect of the drug on CD133^+^ OS-CSC [141]. Another natural derivative with reported antitumoral activity in OS is diallyl trisulfide, which can reverse drug resistance through the downregulation of ABCB1, and, in combination with methotrexate, is able to reduce the CD133^+^ subpopulation of drug resistant human OS cells [142]. Antitumoral effects of diallyl trisulfide seem to be mediated by the upregulation of tumor-suppressive miRNAs associated with an inhibition of NOTCH1 signaling [143]. Additionally, several histone deacetylase inhibitors have demonstrated antitumoral activity in OS, including the induction of differentiation in OS-CSC and the reduction of the metastatic potential [144, 145].

Finally, immunotherapy is an attractive option to target CSC subpopulations. Thus, the treatment of human OS cell lines with T cells expressing a specific chimeric antigen to target the human epidermal growth factor receptor ERBB2 was able to efficiently decrease the sarcosphere formation capacity and the ability to generate OS* in vivo*, suggesting that this immune-based strategy is able to target CSC subpopulations [125]. In addition, the membrane receptor CD47, which plays an important role in the mechanisms of tumor immune escape, has been found overexpressed in OS samples and highly expressed in cell subpopulations expressing the CSC marker CD44. Notably, the blockade of CD47 by specific antibodies suppressed the invasive ability and the metastatic potential of OS cells, suggesting a potential use for these anti-CD47 antibodies in the treatment of OS [146].

It is important to mention that CSC subpopulations are heterogeneous and different subpopulations may exist within a tumor with different genetic alterations. Moreover, these subpopulations are highly dynamic and there are processes of dedifferentiation and phenotype switching which may render CSC resistant to a specific CSC therapy [147]. In this regard, future therapies should combine different treatments to target both non-CSCs and CSCs, and those CSC-specific treatments should target multiple pathways altered in different subsets of CSCs within the tumor. These broader spectrum therapeutic approaches include immune-based treatments and/or therapy targeting tumor microenvironment. In addition, inhibition of transcription factors presenting altered activity offers a promising choice since they are pivotal points in signaling pathways and therefore their inhibition may block several routes involved in tumor progression. In this regard, inhibition of SP1 was able to eliminate CSCs in soft tissue sarcoma models [148].

## 6. Conclusions

In the most likely scenario, OS development is initiated by different cell types along the mesenchymal-osteogenic lineage targeted with relevant oncogenic lesions, like the inactivation of the tumor suppressor genes P53 and RB, and highly influenced by bone microenvironment signals. During tumor evolution, CSC subpopulations emerge after the accumulation of further epigenetic and/or genetic alterations in a subset of tumor cells. During the past decades, chemotherapy for treatment of OS has improved the overall survival for patients significantly. However, despite impressive advances, there are very little novel therapeutic agents that target tumors which are metastatic or refractory to current chemotherapy, creating a real need for the development of more biologically focused treatment regimens. OS represent a heterogeneous type of tumors, for which broader spectrum therapeutic approaches should be proposed. These strategies may include combined targeted therapies, immune-based treatments, and/or therapy targeting tumor microenvironment. Recent studies have highlighted the importance of OS-CSCs, which have been associated with chemoresistance, relapse, and metastasis events. More research aimed towards the characterization of CSC biology and evolution during tumor progression is needed to develop powerful methods of detection and efficient therapies. Targeting the tumor OS-CSCs or disrupting the interaction between OS-CSCs and their bone niche also constitutes a valuable approach, with promising clinical trials ongoing that could yield exciting new therapies for the future.

## Figures and Tables

**Table 1:** Selected clinical trials targeting altered signaling and tumor environment in OS^*∗*^.

Target	Drug	Type of drug	Clinical trial reference number (NCT number)
*Cell membrane receptors*			
ERBB2	Trastuzumab	Monoclonal antibody	NCT00023998
IGF1R	Cixutumumab	Monoclonal antibody	NCT01016015/NCT00831844/NCT01614795/NCT00720174
	RG1507	Monoclonal antibody	NCT00642941
EGFR	ZD1839	Inhibitor	NCT00132158
PDGFR	Erlotinib	Inhibitor	NCT00077454
	Imatinib	Inhibitor	NCT00031915/NCT00030667
PRGFR/VEGFR	Sorafenib	Inhibitor	NCT01804374/NCT00889057/NCT00880542/NCT00330421/ NCT01518413
VEGFR	Pazopanib	Inhibitor	NCT01759303/NCT02357810/NCT01130623/NCT02180867/ NCT01532687/NCT01956669
	Bevacizumab	Monoclonal antibody	NCT00667342
	Endostar (rh-endostatin)	Inhibitor	NCT01002092

*Intracellular signaling *			
mTOR	Everolimus	Inhibitor	NCT01216826
	Ridaforolimus	Inhibitor	NCT00093080/NCT00538239
WNT/*β*-catenin	Curcumin	Inhibitor	NCT00689195

*Niche cells and their signaling *			
Osteoclasts	Zoledronic acid	Bisphosphonate	NCT00691236
	Pamidronate	Bisphosphonate	NCT00586846
RANKL	Denosumab	Monoclonal antibody	NCT02470091
Immune system	T cells expressing GD2	Cells	NCT02107963
	GD2Bi-armed T cells	Cells	NCT02173093
	Anti-GD2	Monoclonal antibody	NCT00743496/NCT02502786
	Stem and natural killer cells	Cells	NCT02409576/NCT01807468
	Mifamurtide	Monocyte/macrophage activator glycopeptide	NCT02441309/NCT00631631

^*∗*^Source: https://clinicaltrials.gov/. Osteosarcoma clinical trials: total: 363, open: 122.

ERBB2: Erb-B2 receptor tyrosine kinase 2; IGF1R: insulin-like growth factor 1 receptor; EGFR: epidermal growth factor receptor; PDGFR: platelet-derived growth factor receptor; VEGFR: vascular endothelial growth factor receptor; mTOR: mechanistic target of rapamycin; WNT: wingless-type MMTV integration site family; RANKL: receptor activator of nuclear factor kappa-B ligand.

**Table 2:** Therapeutic agents with reported activity on OS-CSCs subpopulations or related properties.

Therapeutic agent	Proposed mechanisms of action	Effect on CSC/CSC properties	Reference
Parthenolide	NF-*κ*B inhibition/oxidative stress induction	Sensitizes to ionizing radiation reducing the viability of CD133^+^ CSCs	[31]
BRM270	NF-*κ*B/CDK6/IL6 downregulation	Induces programmed cell death	[32]
BYL719	PI3K inhibition	Induces cell cycle arrest and inhibits migration	[33]
LY294002	PI3K inhibition	Induces cell cycle arrest and apoptosis in OS-sarcospheres	[34]
SB431542	TGF-*β* inhibition	Reduces self-renewal and differentiation and increases chemosensitivity of OS-sarcospheres	[35]
miR-382 expression	YB-1 inhibition	Decreases OS-CSCs, reduces metastatic potential, and inhibits tumor formation from CD133^+^ OS cells	[36]
miR-29b-1 expression	—	Reduces sarcosphere formation and induces chemosensitization of OS cells	[37]
miR-133a inhibition	—	Reduces cell invasion of CD133^+^ OS cells and suppresses metastasis in combination with chemotherapy	[38]
lncRNA HIF2PUT	HIF-2*α*	Reduces CD133^+^ cells and impairs sphere-forming in OS cells	[39]
Metformin	AMPK/mTOR signaling alteration	Reduces sphere-forming ability and sensitizes OS cells to chemotherapeutic agents	[40, 41]
Bufalin	miR-148a/DNMT1/CDKN1B	Inhibits differentiation and proliferation of OS-sarcospheres	[42, 43]
Salinomycin	WNT signaling downregulation	Reduces sphere-formation and tumor-initiation ability of OS cells and sensitizes them to chemotherapeutic drugs	[44]
Salinomycin-loaded nanoparticles	—	When combined with CD133 aptamers selectively targets OS-CD133^+^ cells	[45]
Diallyl trisulfide	Upregulation of tumor-suppressive miRNAs/inhibition of NOTCH1 signaling/downregulation of ABCB1	Prevents invasion, angiogenesis, and drug resistance in OS cells and in combination with methotrexate reduces OS-CD133^+^ cells	[46, 47]
MC1742/MC2625	Histone deacetylase inhibition	Induces apoptosis and promotes differentiation of sarcoma CSCs	[48]
Vorinostat	Histone deacetylase inhibition	Reduces metastatic potential of OS cells	[49]
Anti-CD47 antibody	Increased macrophage phagocytosis	Inhibits invasion and metastasis of OS cells	[50]

CDK6: cyclin-dependent kinase 6; IL6: interleukin 6; TGF-*β*: transforming growth factor *β*; YB-1: Y box-binding protein 1; HIF-2*α*: hypoxia inducible factors 2*α*; AMPK: AMP-activated protein kinase; mTOR: mechanistic target of rapamycin; DNMT1: DNA (cytosine-5-)-methyltransferase 1; CDKN1B: cyclin-dependent kinase inhibitor 1B; WNT: wingless-type MMTV integration site family; ABCB1: ATP-binding cassette subfamily B member 1.
